# Acquisition of optical coherence tomography angiography metrics during hemodialysis procedures: A pilot study

**DOI:** 10.3389/fmed.2022.1057165

**Published:** 2022-12-01

**Authors:** Giuseppe Coppolino, Davide Bolignano, Pierangela Presta, Fausto Francesco Ferrari, Giovanna Lionetti, Massimiliano Borselli, Giorgio Randazzo, Michele Andreucci, Angelica Bonelli, Antonietta Errante, Leonardo Campo, Davide Mauro, Sarah Tripodi, Robert Rejdak, Mario Damiano Toro, Vincenzo Scorcia, Adriano Carnevali

**Affiliations:** ^1^Renal Unit, University “Magna Græcia” of Catanzaro, Catanzaro, Italy; ^2^Department of Ophthalmology, University “Magna Græcia” of Catanzaro, Catanzaro, Italy; ^3^Department of Ophthalmology, Vigevano-Azienda Socio-Sanitaria Territoriale (ASST) Pavia Civil Hospital, Pavia, Italy; ^4^Chair and Department of General and Pediatric Ophthalmology, Medical University of Lublin, Lublin, Poland; ^5^Eye Clinic, Public Health Department, University of Naples Federico II, Naples, Italy

**Keywords:** intradialytic hypotension, hemodialysis, optical coherence tomography angiography, circulation, choroidal vessel

## Abstract

**Background and aims:**

The observation of optical microcirculation gives us an extraordinary way to directly assess *in vivo* the responses of human circulation to stress stimuli. We run a pilot study to analyze optical coherence tomography angiography (OCT-A) metrics at determined time-points during a hemodialysis (HD) session to understand how these metrics gradually change and to evaluate possible correlations with patients’ characteristics.

**Methods:**

After the eligibility screening, 15 patients (23 eyes) were included in the study. OCT-A parameters were collected at established time-points: Before treatment (t0), at first hour (t1), at second hour (t2), at third hour (t3), and finally at the end of HD treatment (t4). Patients were finally shared in hypotensive group if they occurred in a hypotensive episode during subsequent month methods or no hypotensive group. The instrument software automatically segmented OCT-A scans into four en-face slabs: The superficial capillary plexus (SCP), the deep capillary plexus (DCP), the outer retinal plexus and the choriocapillaris plexus. In this study we focus on SCP, DCP plexuses.

**Results:**

Overall, the majority of ophthalmic parameters remained unaffected and comparable at dialysis end; a significant reduction being observed at the end vs. starting of HD only for deep capillary plexus (DCP: Whole, fovea, and parafovea) and for central choroid thickness (CCT) (*p* < 0.05). An overall trend during the session showed in general a decrease with a significance in particular for DCP (whole, fovea, and parafovea) and for CCT (*P* = 0.006). In the hypotension group, Superficial capillary plexus (SCP: Fovea and parafovea) significantly increased comparing post vs. pre-dialysis values while CCT significantly decreased. Analyzing the trend during treatment only CCT maintained a significant trend (*p* for trend = 0.002). In the no-hypotension group, neither pre- vs. post-analysis and trend analysis showed a statistical significance.

**Conclusion:**

Main achievement of our study was to measure, for the first time in literature, single parameters at different time-points of a HD session. As a result of this process we did not notice a brusque decreasing or increasing of OCT-A metrics but we can characterize the different effect of HD on the two distinct areas distinguishing ocular vessels: Retinal and choroidal circulation. As interesting sub-analysis, Hypotensive group showed for CCT a decreasing trend with a difference statistically significant respect to the group with no-hypotension maintaining a constant trend. In our opinion, these results suggest the role of autonomic system on vessel control in patients affected by uremia.

## Introduction

The introduction of optical coherence tomography angiography (OCT-A), a dye-free imaging technique, has made possible to detect the angiographic characteristics of retinal and choroidal structures and to detect changes in capillary network (typical for example of diabetic retinopathy) even before the onset of the disease. The study of microcirculation, in this context, may play a role as indicator of the health of systemic circulation in general. Different studies related retinal and choroid parameters to the coronary circulation demonstrating how they underwent changes even before the appearance of typical symptoms of coronary stenosis. An oculist exam in different experiences allowed to reach an early diagnosis and risk stratification of the cardiopathic patient (1–3). In this context, OCT-A parameters related to ocular microvasculature could be useful to record and understand their answer to physiological and pathophysiological stress stimuli (4). Among these stimuli it was widely investigated hemodialysis (HD) (a technique to substitute blood purification in patients with total loss of renal function) by structural optical coherence tomography (OCT) (5) and by the OCT-A (6). Every study analyzed, with contrasting results, the sudden effect of HD on eyes comparing modifications of parameters before and at the end of the treatment. Some authors evidenced a significant reduction of choroidal thickness (CT) correlating it with the systemic removal of fluid during HD (7) or decreasing of systemic blood pressure (6, 8); conversely other authors like Jung (9) distinguished between a decrease of subfoveal CT and an increase in choroidal extravascular interstitial density. Some others indicated no modifications (10) or even a paradoxical increase (9). In a recently paper (11) we found that the majority of parameters remained unaffected at dialysis end with a substantial trend of decreasing but statistically insignificant while for central choroid thickness (CCT) we recorded a significant reduction. Interestingly in the same study CT was useful to predict the occurrence of hypotension episodes in the subsequent 30 days and we evidenced in accordance with other authors the different pattern of acquired images following dialysis. All studies analyzed ocular metrics before dialysis and then after 4 h (the standard dose for a HD session) without investigating what could happen during the treatment. Starting from these evidences in a pilot prospective study, we aimed at analyzing continuous variations of ocular parameters acquired by OCT-A at determined time-points during the HD session to understand how these metrics gradually change and to evaluate possible correlations with patients’ characteristics.

## Materials and methods

### Patients selection

Hemodialysis patients from the Dialysis Unit of the University Magna Graecia of Catanzaro, Italy were investigated for admissibility to join the study. Methods and eligibility criteria were duplicate with our previous study (11). The study was conducted in agreement with the Declaration of Helsinki for research involving human subjects and was approved by the local institutional review board (Comitato Etico Area Centro, Regione Calabria). An informed consent was acquired from all participants. In brief all subjects were on renal replacement treatment with a rhythm of 4-h sessions/three times a week, had a stable dry-weight for at least 3 months before arriving the study and had attained a normotensive edema-free state. The session consisted of a standard HD using common dialysis solutions, with bicarbonate buffer at a blood flow rate of 300 ml/min and a dialysate flow rate of 500 ml/min. The dialyzer used was a Flexya dialysis monitor (Bellco^®^, Mirandola, Italy). Dialysate sodium concentration was the same for all the patients at 140 mEq/l and dialysate temperature provided at 36.5°C. Mean UF rate for hour never exceeded 0.6 Kg/h. Adequacy of dialysis was assessed using KT/V, calculated as the logarithm of the ratio between initial and final urea concentration. All patients received a bolus (25–30 IU/Kg) of non-fractioned heparin at the start of the dialysis procedure, which was followed by a maintenance hourly dose (500–2,000 U). Weight loss and systolic/diastolic blood pressure (SBP/DBP) were measured for each patient every hour. Exclusion criteria were dialysis vintage <6 months, recent history of hospitalization for cardiovascular diseases and severe cognitive or physical impairment (12, 13). Each patient underwent dilated fundus ophthalmoscopy and ocular exclusion criteria included any other retinal diseases (including retinal vascular diseases, vitreoretinal diseases, or macular dystrophies), any previous eye surgical intervention or laser photocoagulation in the study eye, ocular media opacity, poor quality images with significant artifact, inaccurate or incorrect segmentation at the level of the superficial capillary plexus (SCP) and deep capillary plexus (DCP).

### Optical coherence tomography angiography metrics

**FIGURE 1:**
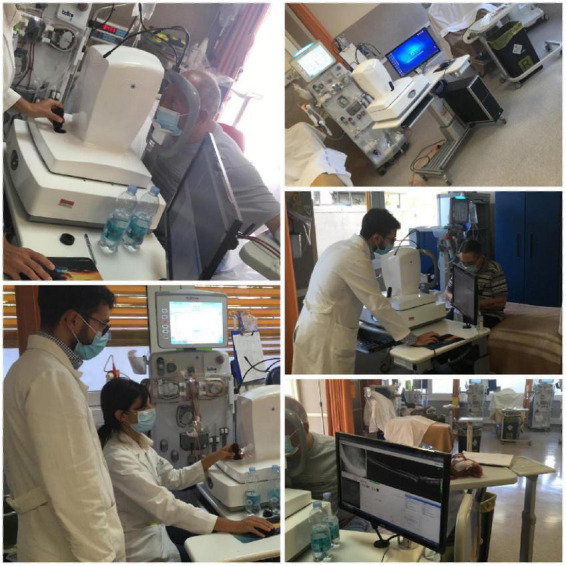
Acquisition of OCT-A parameters during in a haemodialysis session.

**FIGURE 2:**
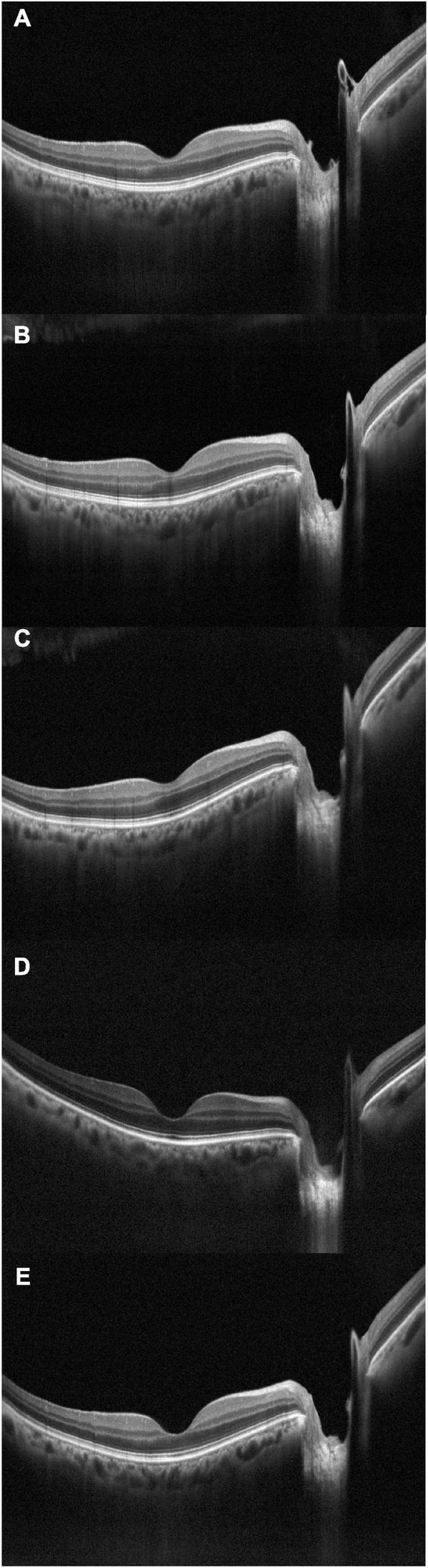
Optical coherence tomography crossline during dialysis, acquired at subsequent times: **(A)** t0, baseline; **(B)** t1; **(C)** t2; **(D)** t3; **(E)** t4.

Measurement of OCT-A metrics was performed in the University Magna Graecia of Catanzaro from the team of the Medical Retina and Imaging Unit of the Department of Ophthalmology. All measurements were performed by qualified ophthalmologists (V.G and A.C.) experts in retinal imaging. Every patient underwent dilated fundus ophthalmoscopy, structural OCT and OCT-A. Patients were seated at the front of the device and informed to fixate the internal fixation light. Measurements were executed at established time-points during the HD session: before treatment (t0), at first hour (t1), at second hour (t2) and at third hour (t3), finally at the end of HD treatment (t4). During HD session patients stayed in sitting position without interfering with HD process (Figures 1, 2). Participants were examined at the last HD session of the week (Friday or Saturday). Ophthalmic exclusion criteria included any other retinal diseases (including retinal vascular diseases, vitreoretinal diseases, history of central serous retinopathy, or macular dystrophies), any previous eye surgical intervention or laser photocoagulation in the study eye, ocular media opacity, inaccurate or incorrect segmentation at the level of the SCP and DCP, or subject’s incapacity to desist from blinking or movement during image gaining, poor quality images with significant artifact. The quality scores of scans were expressed as a signal-to-noise ratio (SNR) in decibels (dB) on a scale of 1 (poor quality) to 40 (excellent quality), and the included scans had a score >20 dB, which was considered good quality.

### Optical coherence tomography and optical coherence tomography angiography image acquisition

Optical coherence tomography angiography was performed using XR Avanti AngioVue OCT-A (Optovue, Fremont, CA, USA). The Optovue instrument uses an A-scan rate of 70,000 scans per second, a light source centered at 840 nm, and a full-width at half maximum (FWHM) bandwidth of 45 nm. The split-spectrum amplitude-decorrelation angiography (SSADA) method was used to capture the dynamic motion of the red blood cells and provides a high-resolution 3D visualization of perfused retinal vasculature. Each scan in this system contains 304 × 304 A-scans with two consecutive B-scans at each fixed position.

We acquired a macular volumetric scan centered on the fovea using the 3 mm × 3 mm and 6 mm × 6 mm scanning area and we collected data from SCP and DCP plexuses. OCT-A images of SCP and DCP networks were detected using the automated software algorithm: The boundaries of superficial network extended from 3 μm below the internal limiting membrane to 15 μm below the inner plexiform layer. A 30-μm-thick layer from the inner plexiform layer was used to visualize the DCP.

Vessel density (VD) of SCP and DCP in the whole image, foveal, and parafoveal zone was automatically calculated as the proportion of measured area occupied by flowing blood vessels defined as pixels having decorrelation values acquired by the SSADA algorithm above the threshold level. The software also calculated automatically the foveal avascular zone (FAZ) area expressed in mm^2^ in the retina plexus.

We collected the following parameters from each patient: SCP VD and DCP VD of whole image, foveal and parafoveal zone and FAZ for both OCT-A 3 mm × 3 mm and 6 mm × 6 mm scans.

Structural OCT was performed using RTVue OCT (Optovue Inc., Fremont, CA, USA), a high-speed and high-resolution spectral domain OCT device with central wavelength of 840 nm, scan rate of 26,000 A-scans/s, and axial resolution of 5 μm. HD line scan, a linear 6 mm high-definition B-scan centered on the fovea at 0°, was used to manually calculate CCT at the fovea: the vertical distance between the hyper scattering retinal pigment epithelium layer and the chorioscleral interface was measured manually using a software caliper built into the custom-made OCT image viewer. HD line scan was used also to calculate the choroidal vascularity index (CVI) using previously reported automated algorithm (14–16). Briefly, the OCT image is opened in ImageJ, and the polygon tool is used to select a region of interest of the entire length of the B-scan (6 mm), centered on the fovea. The upper boundary of the region of interest is traced along the choroidal–RPE junction and the lower boundary along the sclerochoroidal junction to identify the total choroidal area (TCA). Image brightness is adjusted on the base of the average value obtained from the LCA of three choroidal vessels selected using the oval selection tool. After conversion to an 8-bit image, Niblack’s autolocal threshold is applied to binarize the image and to demarcate the LCA and the SCA. The image is converted to a red, green, and blue image, and the color threshold tool is used to select the dark pixels, representing the LCA. The TCA and LCA are finally measured. The SCA is then obtained by subtracting LCA from TCA. The ratio between LCA and TCA is calculated.

All measurements were replicated in both eyes of the same patient and, if successful, a weighted mean of the two values was computed for each parameter and used for analysis.

### Prospective follow-up

After the baseline evaluation, patients were prospectively surveyed up to 30 days and any hypotensive episode occurred through the correspondent 10 dialysis sessions was recorded. Intradialytic hypotension episodes were defined as systolic blood pressure fall during dialysis superior than 20 mmHg or a reduction in mean arterial pressure of 10 mm Hg with symptoms such as headache, nausea, confusion, dizziness, sweating necessitating nurse intervention considering, however, a documented nadir systolic pressure <90 mmHg (17).

### Statistical analyses

The analyses were completed using the SPSS package (version 24.0; IBM corporation, Armonk, New York, NY, United States), the MedCalc Statistical Software (version 14.8.1) and the GraphPad prism software (version 8.4.2, San Diego, California USA). Data were presented as mean ± SD for normally distributed values (at Kolmogorov–Smirnov test), median (IQ range) for variables with skewed distribution or frequency percentage. Differences between groups were determined by the unpaired *T*-test for normally distributed values, the Mann–Whitney U-test for non-parametric values and the chi-square followed by a Fisher’s exact test for frequency distributions. HD variations of retinal parameters were analyzed by a paired-*T*-test or by a Wilcoxon signed-rank test for non-parametric values.

## Results

### Study cohort and baseline assessment

The source population consisted of 35 HD patients. After the eligibility screening 20 patients were excluded because refused to contribute or were not on a regular HD regimen or were excluded due to the occurrence of important retinal or ocular alterations impeding the reliability of measurements in both eyes or because, for technical difficulties, we lost too much time during HD measurements. The final study cohort comprised 15 prevalent patients with a total of 23 eyes being correctly analyzed. The overall intra-patient concordance of measurements between the two eyes was very elevated for all parameters (R ranging from 0.879 to 0.994). Mean age of patients was 64.3 ± 9.2 years and the majority of them were male (66.2%). The median dialysis vintage was 27 months (IQR 16–48). Table 1 summarizes the main cohort data.

**TABLE 1:** Main clinical and laboratory characteristics of the population and differences between subgroups.

	All (*N* = 15)	Hypotension (*N* = 10)	No-hypotension (*N* = 5)	*P*-values
Age (years)	64.3 ± 9.2	**65.2 ± 9.2**	**64.1 ± 10.8**	**0.02**
Gender (% Male)	66.2	65.5	67.5	0.56
Dry-weight (kg)	75.14 ± 11.55	75.8 ± 12.2	75.2 ± 13.2	0.29
Kt/V	1.47 ± 0.25	1.47 ± 0.13	1.47 ± 0.14	0.88
Dialysis vintage (month)	27 (16–48)	28 (16–39)	27 (11–43)	0.78
Diabetes (%)	27.4	29.5	26.5	0.85
History of myocardial ischemia (%)	23.2	25.2	21.3	0.74
Hypertension (%)	71.4	77.3	67.4	0.18
Systolic blood pressure (mmHg)	141.6 ± 7.0	136.7 ± 8.1	144.1 ± 6.1	0.12
Diastolic blood pressure (mmHg)	72.4 ± 14.2	72.4 ± 19.5	72.5 ± 11.4	0.76
Serum phosphate (mg/dL)	5.6 ± 0.67	5.6 ± 0.5	5.7 ± 0.7	0.51
Serum calcium (mg/dL)	8.7 ± 0.83	8.7 ± 0.52	8.7 ± 1.2	0.67
Parathormone (pg/mL)	369 (187.7–494.4)	366 (298–566)	311 (156–398)	0.47
Albumin (g/dL)	3.94 ± 0.35	3.96 ± 0.44	3.94 ± 0.31	0.75
LDL cholesterol (mg/dL)	76.2 ± 31.3	77.1 ± 29.1	75.7 ± 21.7	0.05
Total cholesterol (mg/dL)	142.4 ± 35.9	143.5 ± 31.9	142.4 ± 34.5	0.55
Triglycerides (mg/dL)	125.2 (83.5–157)	119 (84–149)	129 (71.2–169.5)	0.56
Hematocrit (%)	33.7 ± 4.2	33 ± 4.87	33.8 ± 5.7	0.73
Hemoglobin (g/dL)	10.8 ± 0.82	10.9 ± 0.79	10.8 ± 1.37	0.84
White blood cells (*n* × 10^3^)	6.61 ± 2.5	6.8 ± 2.6	6.6 ± 0.4	0.44
Uric acid (mg/dL)	5.9 ± 1.0	5.9 ± 0.76	6.1 ± 1.4	0.59
C-reactive protein (mg/L)	3.7 (3.2–5.7)	3.9 (2.5–6.7)	2.7 (1.2–6.4)	0.35
Ferritin (mg/dL)	262 (122.5–392.5)	285 (194–446)	224 (125–301.5)	0.46
Serum iron (mg/dL)	72.6 ± 36.8	77.1 ± 40.6	66.2 ± 30.6	0.37
b2-microglobulin (mg/L)	26.2 ± 0.6.2	25.9 ± 7.1	26.6 ± 4.7	0.76
Urea (mg/dL)	142.9 ± 31.6	143.2 ± 34.6	142.5 ± 27.7	0.94
Fibrinogen (mg/dL)	331.4 ± 97.1	353.4 ± 86.8	299.6 ± 104.8	0.08

Bold values represent the statistically significant differences.

### Effects of a single hemodialysis session on optical coherence tomography angiography metrics

Generally, the majority of ocular parameters persisted unaffected and comparable at dialysis end (Table 2), a significant decrease being noticed at the end of HD vs. starting of HD only for DCP (whole, fovea, and parafovea) and for CCT (*p* < 0.05). An overall trend during the HD session showed in general a decrease with a statistical significance in particular for DCP (whole, fovea, and parafovea) and for CCT (*P* = 0.006). The average weight loss at dialysis end was significantly decreased (*P* = 0.05) with a significant decreasing trend (*P* = 0.00).

**TABLE 2:** Dialysis changes in OCT-A metrics in the whole cohort.

Variable	t0 (pre-HD)	t1	t2	t3	t4 (post-HD)	*	#
**SCP**							
Whole	40.71 ± 5.13	40.08 ± 5.78	40.87 ± 5.00	41.03 ± 5.16	41.42 ± 3.14	0.45	0.52
Fovea	16.06 ± 6.85	16.30 ± 7.33	20.03 ± 8.75	18.50 ± 7.48	17.16 ± 7.03	**0.07**	0.18
Parafovea	43.40 ± 5.99	42.67 ± 6.06	42.08 ± 8.99	43.89 ± 5.43	44.08 ± 3.44	0.50	0.16
**DCP**							
Whole	47.76 ± 5.07	46.72 ± 4.48	47.64 ± 3.71	46.83 ± 4.23	42.82 ± 10.19	**0.02**	**0.05**
Fovea	32.26 ± 8.31	31.60 ± 9.25	33.54 ± 7.93	33.69 ± 7.47	30.59 ± 6.87	**0.03**	**0.02**
Parafovea	50.03 ± 5.44	49.24 ± 4.50	50.13 ± 3.67	49.08 ± 4.77	47.34 ± 3.84	**0.01**	**0.002**
CVI	0.64 ± 0.03	0.63 ± 0.04	0.64 ± 0.04	0.63 ± 0.02	0.64 ± 0.03	0.32	0.92
FAZ	0.28 ± 0.11	0.27 ± 0.11	0.25 ± 0.11	0.25 ± 0.12	0.27 ± 0.12	0.30	**0.09**
CCT	193.83 ± 43.88	193.00 ± 47.62	191.55 ± 46.14	175.33 ± 49.00	181.82 ± 47.77	**0.05**	**0.006**
Weight (Kg)	77,12 ± 12,32	76,63 ± 12,17	75,99 ± 11,96	77,45 ± 10,02	75,14 ± 11,55	**0.00**	**0.05**

Pre- and post *T*-test comparing and trend analysis. Statistically significant differences are shown in bold. *P-value Pre-HD vs. Post-HD. ^#^*P*-value for trend. CRT, central retinal thickness; FAZ, foveal avascular zone; DCP, deep capillary plexus; SCP, superficial capillary plexus; CVI, choroidal vascularity index. Bold values represent the statistically significant differences.

### Prospective follow-up and intradialytic hypotension

Throughout the follow-up of 30 days, 77 hypotensive episodes corresponding to a frequency rate of 2.35 episodes/pts/month (95% CI 2.6–3.48) were registered. Each hypotension event was resolved with appropriate therapy and no clinical sequelae. Patients shared in two groups: Hypotensive group if they occurred in a hypotensive episode as criteria described in methods or no hypotensive group. Ten patients were included in hypotension group with a mean of 5.60 ± 2.22 episodes in the following month while five patients were included in no hypotensive group.

**FIGURE 3:**
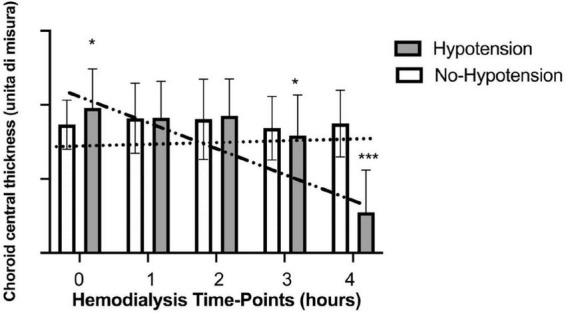
Variations of choroid central thickness during haemodialysis in hypotensive and no hypotensive group. *Statistically significant differences. ***Strong statistically significant differences.

In the hypotension group SCP (fovea and parafovea) significantly increased comparing post- vs. pre-dialysis values while CCT significantly decreased (Table 3). Analyzing the trend during treatment only CCT maintained a significant trend (*p* for trend = 0.002) (Figure 3). In the no-hypotension group neither pre- vs. post-analysis and trend analysis showed a statistical significance (Table 4).

**TABLE 3:** Dialysis changes in OCT-A metrics in the hypotension-group.

Variable	Hypotension					
	Whole	Fovea	Parafovea	CVI	FAZ	CCT
**SCP**						
t0 (pre-HD)	40.81 ± 5.47	12.09 ± 2.50*	43.96 ± 6.72*	0.64 ± 0.03	0.32 ± 0.11	195.90 ± 52.42*
t1	37.96 ± 5.39	12.07 ± 3.26	40.63 ± 6.26	0.62 ± 0.03	0.34 ± 0.10	182.56 ± 49.29
t2	39.05 ± 3.44	15.89 ± 8.73	42.20 ± 4.28	0.63 ± 0.03	0.30 ± 0.15	185.00 ± 49.82
t3	40.08 ± 6.18	14.12 ± 3.12	43.05 ± 7.13	0.63 ± 0.02	0.36 ± 0.12	158.50 ± 54.88
t4 (post-HD)	41.20 ± 3.31	13.38 ± 3.24	44.07 ± 4.18	0.64 ± 0.03	0.34 ± 0.15	170.22 ± 56.87
**DCP**						
t0 (pre-HD)	45.12 ± 5.22	25.75 ± 4.36	47.48 ± 5.57			
t1	46.28 ± 5.00	25.91 ± 3.97	49.41 ± 5.36			
t2	46.05 ± 4.32	29.06 ± 9.04	49.41 ± 4.08			
t3	46.48 ± 5.53	27.05 ± 3.66	50.02 ± 6.45			
t4 (post-HD)	43.64 ± 3.05	25.07 ± 3.79	46.86 ± 2.46			

Pre- and post *T*-test comparing. Statistically significant differences are shown with asterisks (*). CRT, central retinal thickness; FAZ, foveal avascular zone; DCP, deep capillary plexus; SCP, superficial capillary plexus; CVI, choroidal vascularity index. Bold values represent the statistically significant differences.

**TABLE 4:** Dialysis changes in OCT-A metrics in the no-hypotension-group.

	No-hypotension					
	Whole	Fovea	Parafovea	CVI	FAZ	CCT
**SCP**						
t0 (pre-HD)	41.28 ± 5.86	16.54 ± 5.24	43.70 ± 6.68	0.63 ± 0.02	0.30 ± 0.09	173.25 ± 33.04
t1	40.16 ± 5.83	15.85 ± 5.28	42.59 ± 5.96	0.65 ± 0.04	0.22 ± 0.06	181.88 ± 47.25
t2	41.76 ± 4.11	22.03 ± 7.23	39.87 ± 13.25	0.63 ± 0.02	0.23 ± 0.06	180.57 ± 54.08
t3	40.47 ± 4.83	17.78 ± 5.31	43.35 ± 4.42	0.64 ± 0.02	0.20 ± 0.05	168.50 ± 42.77
t4 (post-HD)	40.89 ± 3.53	16.49 ± 5.77	43.48 ± 3.57	0.64 ± 0.02	0.22 ± 0.06	174.75 ± 45.18
**DCP**						
t0 (pre-HD)	49.49 ± 3.92	35.44 ± 6.88	52.03 ± 3.79			
t1	46.46 ± 4.69	31.85 ± 9.23	48.63 ± 4.28			
t2	48.97 ± 3.26	35.10 ± 4.58	51.01 ± 3.36			
t3	47.27 ± 3.82	36.03 ± 4.92	48.95 ± 3.46			
t4 (post-HD)	40.90 ± 16.94	33.21 ± 5.42	48.88 ± 4.61			

Pre- and post *T*-test comparing (no statistically significant differences were found). CRT, central retinal thickness; FAZ, foveal avascular zone; DCP, deep capillary plexus; SCP, superficial capillary plexus; CVI, choroidal vascularity index. Bold values represent the statistically significant differences.

## Discussion

In the present study we explored the reaction of ocular vascular structures to HD treatment by OCT-A, a non-invasive and reproducible tool becoming more and more widespread in ophthalmologic ambulatories. Main achievement was to illustrate and to measure, for the first time in literature, single parameters at different time-points of a HD session. In all other experiences, until now, only single acquisitions have been conducted before and after 4 h of HD. For this reason, we were unaware of vascular adaptations of OCT-A metrics during the prolonged time of HD. HD is a technique for substitution of renal function in patients on end stage of renal disease (ESRD), using a blood filtration system able to eliminate toxic molecules and to equilibrate hydro-electrolytical alterations. In this process it acts with a dynamic pathway on vascular system. In fact, it removes exceeding water volume and changes numerous metabolic parameters together (for example sodium, potassium, calcium, urea, and others on). The gradual removal of fluid and electrolytes from vessels lumen, starting suddenly with the beginning of the session, leads to a continuous intravascular increase of colloid osmotic pressure and a steady flow of new fluid from interstitial space through fenestrated wall of endothelial line in the vascular wall. The gradient is constantly re-balanced with a movement of water and salts and a remodeling of vessel volume. After this endless sequence of hydro-electrolytical turbulences on cells “membranes and vessels” dynamic, carried on for 4 h, a new equilibrium between interstitial environment and intravascular lumen is found at the end of HD. In general, as a result of this process we did not notice a brusque and high significative decreasing or increasing of OCT-A metrics but we can characterize the different effect of HD on the two distinct areas distinguishing ocular vessels: The retinal and the choroidal circulation. Retinal vessels are notoriously not innervated by the autonomous nervous system and their vascular reactivity is influenced by endothelial function and lumen adaptation to blood stream. In our analysis retinal parameters smoothly adapted to fluid removal resulting in a not statistically significant shift of vessels in SCP and in a slightly significant decreasing in DCP. Conversely choroidal central thickness decreased from starting to end of HD attaining a marked statistical significance (*P* = 0.006). Choroidal vessels are defined as the organ with the highest blood supply per area in the human body with a specific extrinsic autonomic regulation. As interesting sub-analysis, we divided patients basing on the occurrence of hypotension, a common symptom during HD. Hypotension during HD is recognized to be intricate and multifactorial, as it may depend either from a too much aggressive fluid removal with HD but also from unbalanced peripheral and neurohormonal reactions to circulating volume reduction. For minimizing the effect of an excessive fluid removal in all patients we maintained a slow and constant water removal (0.6 Kg/h) and we followed patients for the following 30 days recording hypotensive episodes. It resulted in two small groups: Hypotensive and no-hypotensive. Hypotensive group showed for choroidal central thickness a decreasing trend with a difference statistically significant respect to the group with no-hypotension that maintained a constant trend. In our opinion these results suggest, in accordance with our previous paper (11), the role of autonomic system on vessel control in patients affected by uremia.

A general important aspect of our study was the homogeneity of the cohort and a prospective follow-up to record hypotension episodes. The main strength is, of course, the acquisition of data during the HD session at the determined time-points. Furthermore, differently from other studies reporting acquisitions before and after dialysis, in our cohort parameters were really taken at start and finish of HD and they were not influenced by the time lag to reach the ophthalmology ambulatory. Conversely our study design posed great difficulties because we adapted an OCT-A device commonly used for ambulatory visits and we tailored it to acquire images at the bed of the patient. These aspects created a certain discomfort and stress for the patient producing many missing cases for the refuse of someone to continue the exam or for the loss of acquisitions metrics at determined time-points. The main limit is represented by the little, single-center assessment and the study protocol included measurements in only one dialysis per patient and basic systemic disease possible interfering with OCT-A metrics. However, despite the restricted amount of cases evaluated, we reported a significant number of patients facing IDHs and a relatively great absolute number of episodes over the follow-up period, which allowed to discover substantial associations between retinal parameters and hypotensive episodes. We hypothesized it could be related to autonomic dysregulation but we did not collected specific markers of autonomic nervous system.

## Conclusion

In conclusion the cooperation of ophthalmologists and internists still enjoys, nowadays, of the characterization of damage in retinal vessels through a *fundus oculi* analysis and OCT-A. Visualization of this network of capillaries in systemic diseases permits to have an ideal photographic frame of the vascular status in other organs by a non-invasive and reproducible tool, OCT-A will become a very useful tool for understanding the pathophysiology of systemic vascular diseases and monitoring patients as dialysis.

## Data availability statement

The raw data supporting the conclusions of this article will be made available by the authors, without undue reservation.

## Ethics statement

The studies involving human participants were reviewed and approved by the Comitato Etico Area Centro, Calabria. The patients/participants provided their written informed consent to participate in this study. Written informed consent was obtained from the individuals for the publication of any potentially identifiable images or data included in this article.

## Author contributions

All authors listed have made a substantial, direct, and intellectual contribution to the work, and approved it for publication.
